# Clinical comparison of three-dimensional exoscope vs. operative microscope in transforaminal lumbar interbody fusion: A retrospective case-control study

**DOI:** 10.3389/fsurg.2022.926329

**Published:** 2022-07-29

**Authors:** Yu-jian Peng, Tian-bu Zhao, Jun Dai, Qian-liang Wang, Qian-zhong-yi Zhang, Jun-yin Cao, Xiao-feng Liu

**Affiliations:** ^1^ Department of Orthopedic Surgery, The Second Affiliated Hospital of Soochow University, Suzhou, China; ^2^ Department of Emergency Surgery, The Second Affiliated Hospital of Soochow University, Suzhou, China

**Keywords:** three-dimensional exoscope, transforaminal lumbar interbody fusion (TILF), hidden blood loss (HBL), case-control study, interverbal disc degeneration

## Abstract

**Purpose:**

Here, we sought to determine the safety and feasibility of three-dimensional exoscope (3D EX). We compared data on surgery, complications, postoperative drainage, hematology, and clinical outcomes in the group that underwent transforaminal lumbar interbody fusion (TLIF) using an operative microscope (OM) relative to those treated using 3D EX.

**Methods:**

We retrospectively reviewed records on 74 patients who underwent one- or two-level TLIF from August 2019 to October 2020. Repeated measures analysis of variance was used to compare pre- and post-operative visual analogue scale (VAS) scores and oswestry disability index (ODI). We used analysis of covariance to compare pre- and post-operative erythrocyte count (RBC), hemoglobin (Hb), and hematocrit (Hct). Independent sample t-tests was used to compare postoperative drainage volume, total blood loss (TBL), visible blood loss (VBL) and hidden blood loss (HBL).

**Results:**

There were no significant differences in VAS and ODI scores between the two groups at all time points (all *p *> 0.05). RBC and HBL did not differ significantly between the two groups (all *p *> 0.05). There were significant differences in postoperative drainage volume,TBL, Hb, and Hct values when using 3D EX relative to OM in two-level TLIF (all *p *< 0.05), but not for one-level TLIF (all *p *> 0.05). The two groups differed significantly with regards to VBL when used for one- or two-level TLIF (all *p *< 0.05).

**Conclusion:**

Our data show that 3D EX is a suitable alternative to OM in TLIF. Relative to OM, 3D EX has important strengths in reducing perioperative bleeding in two-level TLIF.

## Introduction

Intervertebral disc degeneration (IDD), a major cause of degenerative spinal disease, severely affects quality of life in elderly populations. Globally, about 266 million people develop degenerative spine disease annually, which poses significant socioeconomic burden in developed countries (1, 2). Spinal fusion is the standard treatment for painful IDD after failed conservative treatment (3). Transforaminal lumbar interbody fusion (TLIF) is currently the main surgical procedure for IDD treatment (4). The introduction of the operative microscope (OM) as a visualization tool for TLIF procedures represented significant advancement. OM allows clear, magnified visualization of anatomical structures, thereby avoiding injury to nerves and blood vessels. With a focal length of 200–415 mm, OM provides space for microsurgical instruments to be placed in the operating space.

Despite its advantages, OM has drawbacks, including a lack of ergonomic design and extreme microscope positional angles needed for successful decompression, especially the contralateral aspect. These compromise the surgeons’ neutral posture, forcing them into a non-ergonomic posture (5). This is even worse for assistants who must adapt to the microscope height set by the operator. The exoscope attempts to overcome the limitations of OM. However, it is reported that compared with OM, hand-eye coordination is clearly affected in the traditional two-dimensional exoscope (2D EX) due to limited depth perception (6).

To overcome the shortcomings of OM and 2D EX, a three-dimensional exoscope (3D EX) has been developed. Several 3D EX systems are currently available for neurosurgery, including the VITOM® 3D (Karl Storz SE & Co. KG, Tuttlingen, Germany), KINEVO® (Carl Zeiss Meditec AG, Oberkochen, Germany), ModusV™ (Synaptive Medical, Toronto, Canada), and ORBEYE™ (Olympus, Tokyo, Japan) (7). Relative to 3D EX mentioned above, the MITAKA KV II (Mitaka Co, Tokyo, Japan) has greater working distance and higher max magnification. 3D EX produces high-quality images with a field of wide and a focal distance of 300–1,000 mm. The high focal distance offers a larger workspace for spine surgery. Because visualizations are displayed on large digital monitors, independence from the eyepiece allows the surgeon and assistant to work ergonomically without contorting their posture (8, 9). Moreover, the 3D technique overcomes the lack of stereoscopic visualization (10). Past studies have mainly focused on the ergonomic advantage of 3D EX (8, 11, 12). Here, we compared data on surgery, complications, postoperative drainage, hematological parameters, and clinical outcomes in patients who underwent TLIF treatment with OM relative to those treated using 3D EX TLIF. To our knowledge, this is the first study to examine the effect of 3D EX on patients undergoing TLIF.

## Materials and methods

### Clinical materials

Ethical approval for this retrospective study was granted by the ethics committee of the Second Affiliated Hospital of Soochow University (approval No. JD-HG-2021-13). Patients were eligible if they underwent a one- or two- level TLIF between L1−S1 for a symptomatic lumbar spinal stenosis or lumbar spondylolisthesis, and they had both complete data. Bilateral decompression was performed in all patients. All surgeries were completed by the same senior surgeons in our hospital. The exclusion criteria were as follows: (1) previous lumbar surgery、infections and tumors (2) antiplatelet or anticoagulant drugs (3) hematological malignancies, bleeding disorders, chronic liver disease. (4) unilateral decompression. (5) patients with missing data. Finally, 74 patients were enrolled in the study. Two groups were formed according to the type of microscope. 3D EX group consisted of 32 patients and OM group consisted of 42 patients.

### Exoscope equipment and operating room set-up

**Figure 1:**
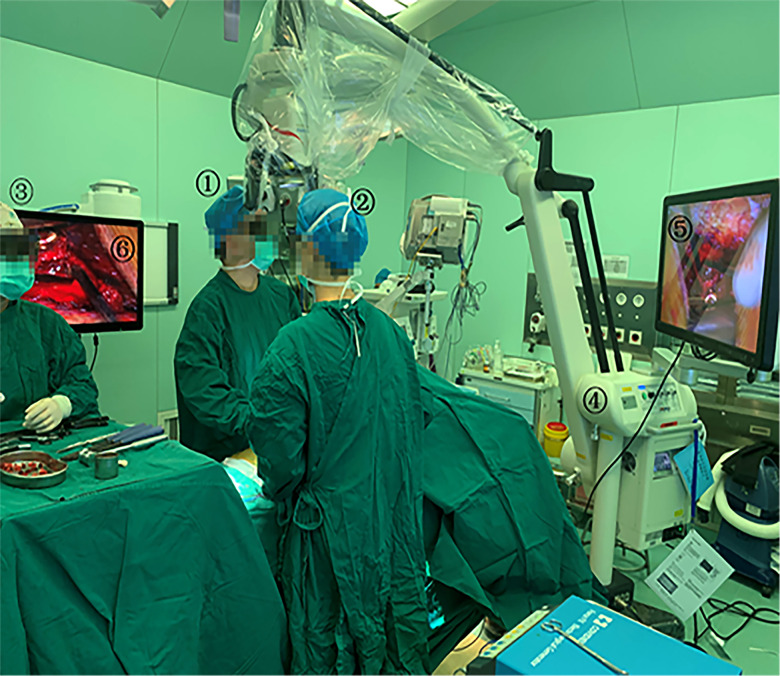
Operating room set-up for 3D EX. 1: Surgeon; 2: Assistant; 3: Scrub nurse; 4: 3D EX(MITAKA KV II); 5 and 6: The 3D video monitor.

The 3D EX (MITAKA KV II, Mitaka Co,Tokyo, Japan) consists of an exoscope, video recorder, light source, and stereoscopic monitor. Compared to a standard OM (S88, ZEISS Inc, Oberkochen, Germany), the 3D EX has longer working distance, wider field of view, and higher magnification. The general parameters of the two different equipment are shown in Table 1. The surgeon uses 3D glasses to see the 3D image on the stereoscopic monitor. The 3D video monitor is best when positioned on the opposite side of the operating table, to the right of the assistant. A second monitor, in a similar position just behind and to the right of the surgeon, offers the assistant an identical field of view (Figure 1).

**Table 1:** Compare the general parameters of the two different microscopes.

	3D EX (MITAKA KV II)	OM (S88)
Working distance(mm)	300–1000	200–415
Zoom ratio	1:8	1:6
Maximum magnification	×40	×13
Resolution(line pairs)	58	56

3D EX, the three-dimensional exoscope; OM, the operating microscope.

### Surgical technique

**Figure 2:**
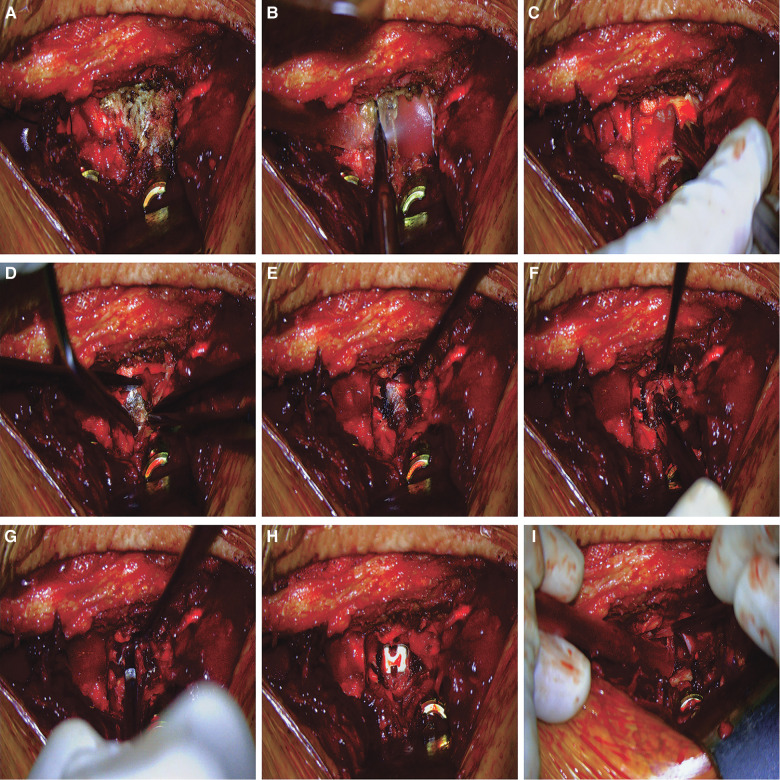
Surgical procedures of 3D EX TLIF. (**A**) Facet joint and lamina were exposed. (**B**) The ultrasonic bone scalpel was used in a unilateral laminectomy and inferior facetectomy. (**C**) The ligamentum flavum was removed with forceps to expose dural sac. (**D**) The epidural veins required coagulation with bipolar cautery. (**E**) Exposure of the disc space. (**F**). Discectomy was performed when a soft herniated disc was identified. (**G**) Endplate preparation. (**H**) A cage was filled with bone chips and inserted into the central part of the disc space. (**I**) Decompression of the neural structures was verified by a micro-hook.

The decompression process in the 3D EX group (from facetectomy to cage placement) were carried out and recorded with the assistance of the EX. Patients were placed in the prone position after general anesthesia. The posterior elements of the spine were exposed to the bases of the transverse processes through a midline longitudinal incision. Pedicle screws were placed into the upper and subjacent vertebral pedicle of the segmental lesions. Facet joint and lamina were exposed (Figure 2A). An ultrasonic bone scalpel was used in a unilateral laminectomy and inferior facetectomy (Figure 2B). The ligamentum flavum was removed with forceps to expose dural sac and the lateral margin of the ipsilateral nerve root (Figure 2C). At this point, the epidural veins required coagulation with bipolar cautery (Figure 2D). After good exposure of the intervertebral space (Figure 2E), discectomy was performed when a soft herniated disc was identified (Figure 2F). After appropriate endplate preparation (Figure 2G), a cage was filled with bone chips and inserted into the central part of the disc space (Figure 2H). Decompression of the neural structures was verified by a micro-hook (Figure 2I). Finally, both rods were mounted under slight compression. In the OM group, microscopic TLIF was performed as described by Harms and Jes-zenszky et al. (13). Surgeries were performed by the same team consisting of three experienced spine surgeons in our hospital.

### Outcome measures

#### Drainage evaluation

Two negative pressure drainage tubes were placed before wound closure. The drain was removed when its output reached ≤50 ml in 24 h, irrespective of drainage duration. Drainages were emptied between 6 and 7AM, each working day. Drainage volume was recorded each day after surgery until drainage tube removal and the total drainage volume determined by summing daily volumes. Drainage tube removal time was also recorded.

#### Perioperative blood loss and hematological evaluation

Preoperative blood routine tests were done within a week before operation. Because 48–72 h were allowed for hemodynamic stabilization, postoperative blood routine tests were done during the recovery period, 2–3 days after surgery.

To calculate total blood loss, we first estimated total blood volume (TBV) using the Nadler formula (14) as follows: TBV = k1 × height (m^3^)+k2 × weight (kg)+k3. For men: k1 = 0.3669, k2 = 0.03219, and k3 = 0.6041. For women: k1 = 0.3561, k2 = 0.03308, and k3 = 0.1833. We used total blood loss (TBL) calculated using Gross’ formula (15) as follows: TBL = TBV × (Hct_Preop_-Hct_Postop_)/Hct_ave_ (Hct_Preop_: hematocrit, Hct, before the operation; Hct_Postop_ = HCT value after operation; Hct_ave_ = average Hct_Preop_ and the Hct_Postop_). If either reinfusion or allogeneic RBC transfusion was performed, the TBL formula was calculated as follows: TBL = TBV×(Hct_Preop_−Hct_Postop_)/Hct_ave _+ Blood infusion. Decisions on whether red blood cell (RBC) transfusion was necessary or not were made by surgeons during surgery, or by attending physicians after surgery. Patients with hemoglobin (Hb) levels of <8.0 g/dl or who were symptomatic received postoperative transfusion. Visible blood loss (VBL) was calculated using the formula: VBL = intraoperative blood loss + postoperative drainage. Hidden blood loss was calculated using the formula: HBL = total blood loss - visible blood loss.

#### Evaluation of clinical outcomes

Clinical outcomes were assessed using visual analogue scale (VAS) scores for back and leg pain, and the Oswestry disability index (ODI). ODI and VAS scores were taken preoperation, 1 week, 1 month, 3 months, 6 months, and 12 months after surgery. Postoperative complications including dural tear and revision were watched and evaluated.

### Statistical analysis

All statistical analyses were done using SPSS version 20.0 (IBM). Enumeration data are expressed in percentage. Measurement data are expressed as mean ± SD. Unless stated otherwise, independent-sample t test was used for measurement data. Chi-square and Fisher exact tests were used for enumeration data. Analysis of covariance was used to compare pre- and post-operative RBC, Hb, and Hct. VAS and ODI were analyzed using general linear models repeated measures analysis of variance. *P *< 0.05 indicated statistically significant differences.

## Results

### Characteristics of the participants at baseline

A total of 74 patients met the inclusion criteria. Of these, 32 were treated using 3D EX, with 20 undergoing one-level TLIF, and 12 undergoing two-level TLIF. OM was used to treat 42 patients, with 23 undergoing one-level TLIF and 19 undergoing two-level TLIF. Of the patients who underwent 3D EX, 30 were diagnosed using lumbar spinal stenosis, while 2 were diagnosed using lumbar spondylosis. Of those treated using OM, 40 were diagnosed using lumbar spinal stenosis, while 2 were diagnosed using lumbar spondylosis. There were no significant differences in the demographics of the two groups. The participants main clinical features are shown on Table 2.

**Table 2:** Patient baseline demographic characteristics.

Variables	OM	3D EX	*p*-value
One-level	23	20	
Surgical level			
L2-L3	0 (0.00%)	2 (10.00%)	0.116
L3-L4	0 (0.00%)	2 (10.00%)	
L4-L5	21 (91.30%)	13 (65.00%)	
L5-S1	2 (8.70%)	3 (15.00%)	
Age, year	60.30 ± 9.25	61.30 ± 10.22	0.739
Sex			
Male	8 (34.78%)	10 (50.00%)	0.313
Female	15 (65.22%)	10 (50.00%)	
Height, m	1.62 ± 0.09	1.63 ± 0.08	0.734
Weight, kg	61.50 ± 10.49	65.88 ± 11.64	0.202
BMI, kg/m^2^	23.41 ± 2.87	24.72 ± 2.91	0.145
ASA score			
I	9	8	0.749
II	13	10	
III	1	2	
Disease			
Lumbar spinal stenosis	22 (95.65%)	18 (90.00%)	0.590
Lumbar spondylolisthesis	1 (4.35%)	2 (10.00%)	
Two-level	19	12	
Surgical level			
L2-L3, L3-L4	1 (5.26%)	1 (8.33%)	0.764
L3-L4, L4-L5	6 (31.58%)	5 (41.67%)	
L4-L5, L5-S1	12 (63.16%)	6 (50.00%)	
Age, year	61.11 ± 13.04	60.83 ± 6.28	0.947
Sex			
male	10 (52.63%)	7 (58.33%)	1.000
female	9 (47.37%)	5 (41.67%)	
Height, m	165 ± 0.07	1.67 ± 0.10	0.514
Weight, kg	67.16 ± 9.10	72.25 ± 11.24	0.176
BMI	24.52 ± 4.99	25.84 ± 4.24	0.250
ASA score			
I	8	4	0.862
II	10	7	
III	1	1	
Disease			
Lumbar spinal stenosis	18 (94.74%)	12 (100.00%)	1.000
Lumbar spondylolisthesis	1 (5.26%)	0 (0.00%)	

Continuous variables were expressed as mean ± SD, Categorical variables were expressed as percentage (%). BMI, body mass index; ASA score, American society of anesthesiologists classification score; 3D EX: the three-dimensional exoscope; OM: the operating microscope.

### Surgical data and related postoperative complications

The two groups did not differ significantly with regards to operative time, intraoperative blood loss, blood infusion volume and postoperative hospital stay. There was statistically significant difference in the use of shed autologous blood reinfusion in the 3D EX vs. OM in two-level TLIF (*p *= 0.035) but not in one-level TLIF (*p *= 1.000). The rate of postoperative complications did not differ significantly between the two groups. Surgical data and associated postoperative complications are summarized on Table 3.

**Table 3:** The surgical data and related postoperative complications.

	OM	3D EX	*p*-value
One-level	23	20	
Operation time, min	138.61 ± 28.31	145.00 ± 31.58	0.488
Intraoperative blood loss, ml	130.43 ± 55.88	114.50 ± 63.62	0.387
Blood transfusion, *n*	0 (0.00%)	2 (10.00%)	0.210
Shed autologous blood reinfusion, *n*	1 (4.34%)	0 (0.00%)	1.000
Blood infusion volume, ml	200.00 ± 0.00	250.00 ± 70.71	0.667
Postoperative hospital stay, day	7.39 ± 2.55	6.40 ± 1.67	0.135
Complications			
Dural tear	2 (8.70%)	0 (0.00%)	0.491
Infection	0 (0.00%)	0 (0.00%)	1.000
Two-level	19	12	
Operation time, min	168.42 ± 43.11	171.67 ± 29.18	0.820
Intraoperative blood loss, ml	255.26 ± 114.13	229.17 ± 65.57	0.426
Blood transfusion, n	0 (0.00%)	0 (0.00%)	1.000
Shed autologous blood reinfusion, n	15 (78.95%)	5 (41.67%)	0.035*
Blood infusion volume, ml	102.40 ± 65.51	97.60 ± 55.78	0.885
Postoperative hospital stay, day	7.05 ± 1.68	6.42 ± 1.00	0.247
Complications			
Dural tear	0 (0.00%)	0 (0.00%)	1.000
Revision	0 (0.00%)	0 (0.00%)	1.000

Continuous variables were expressed as mean ±SD, Categorical variables were expressed as percentage (%). 3D EX, the three-dimensional exoscope; OM, the operating microscope, *p < 0.05.

### Drainage data results

**Figure 3:**
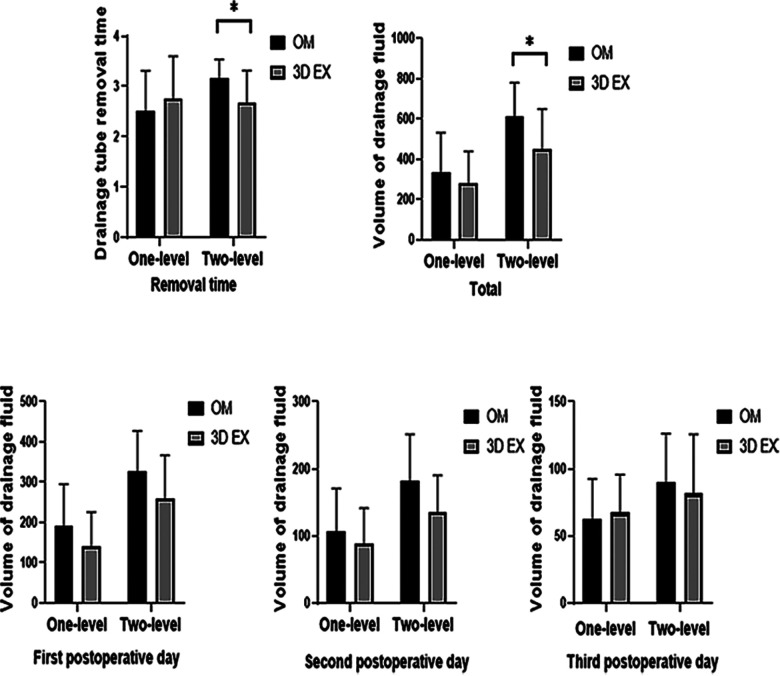
Drainage data results. 3D EX, the three-dimensional exoscope; OM, the operating microscope; *p < 0.05; **p < 0.001.

Drainage fluid volume on the 1st, 2nd, or 3rd day after operation did not differ between the two groups. The mean total drainage fluid volume and drainage tube removal time differed significantly between the 3D EX and OM groups in two-level TLIF (*p *= 0.021, *p *= 0.012) but not in one-level TLIF (*p *= 0.313, *p *= 0.367). Drainage data are shown on Figure 3.

### Perioperative blood loss and hematological results

**Figure 4:**
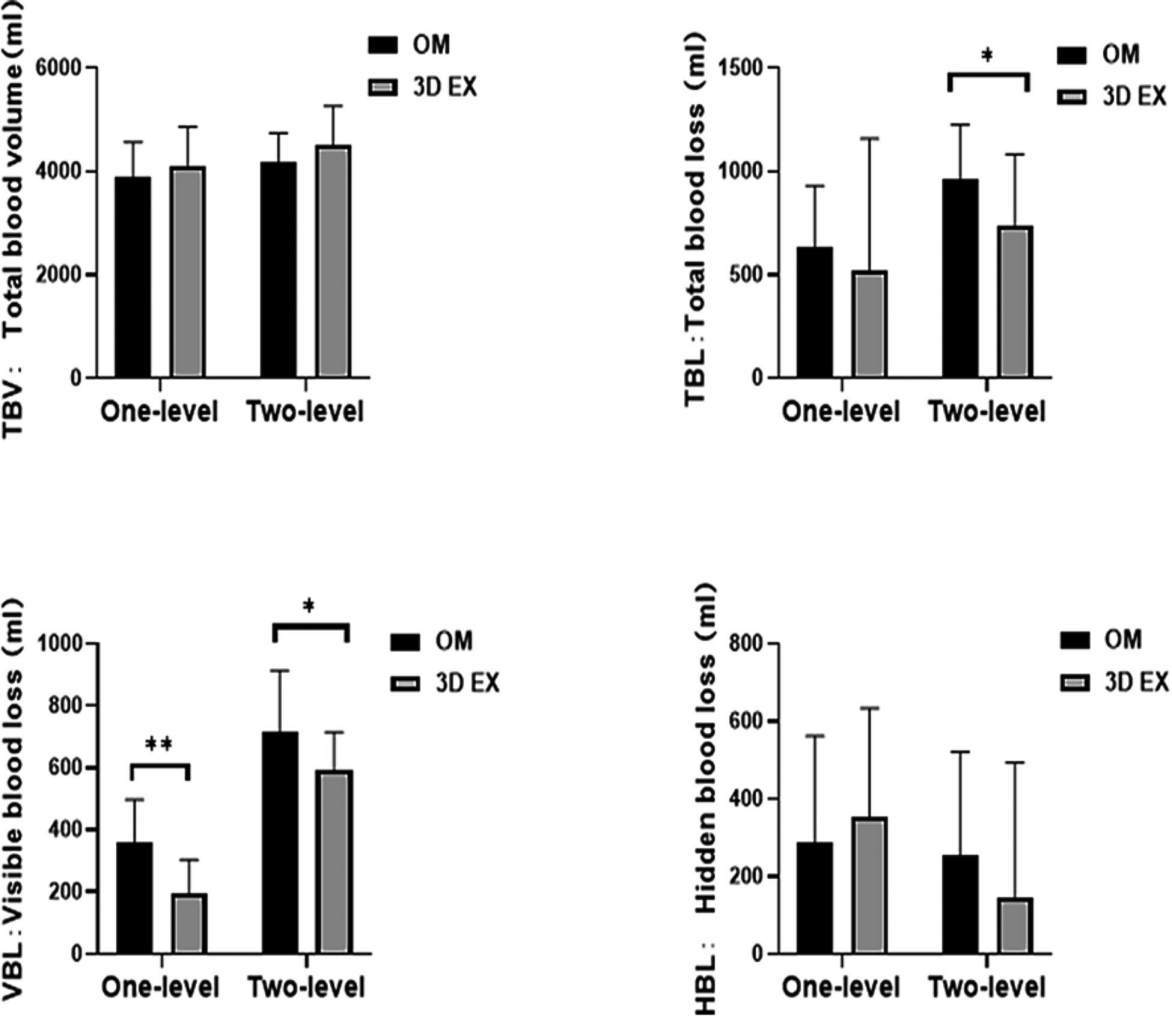
Perioperative blood loss results. 3D EX, the three-dimensional exoscope; OM, the operating microscope; *p < 0.05; **p < 0.01; ns not statistic difference.

**Figure 5:**
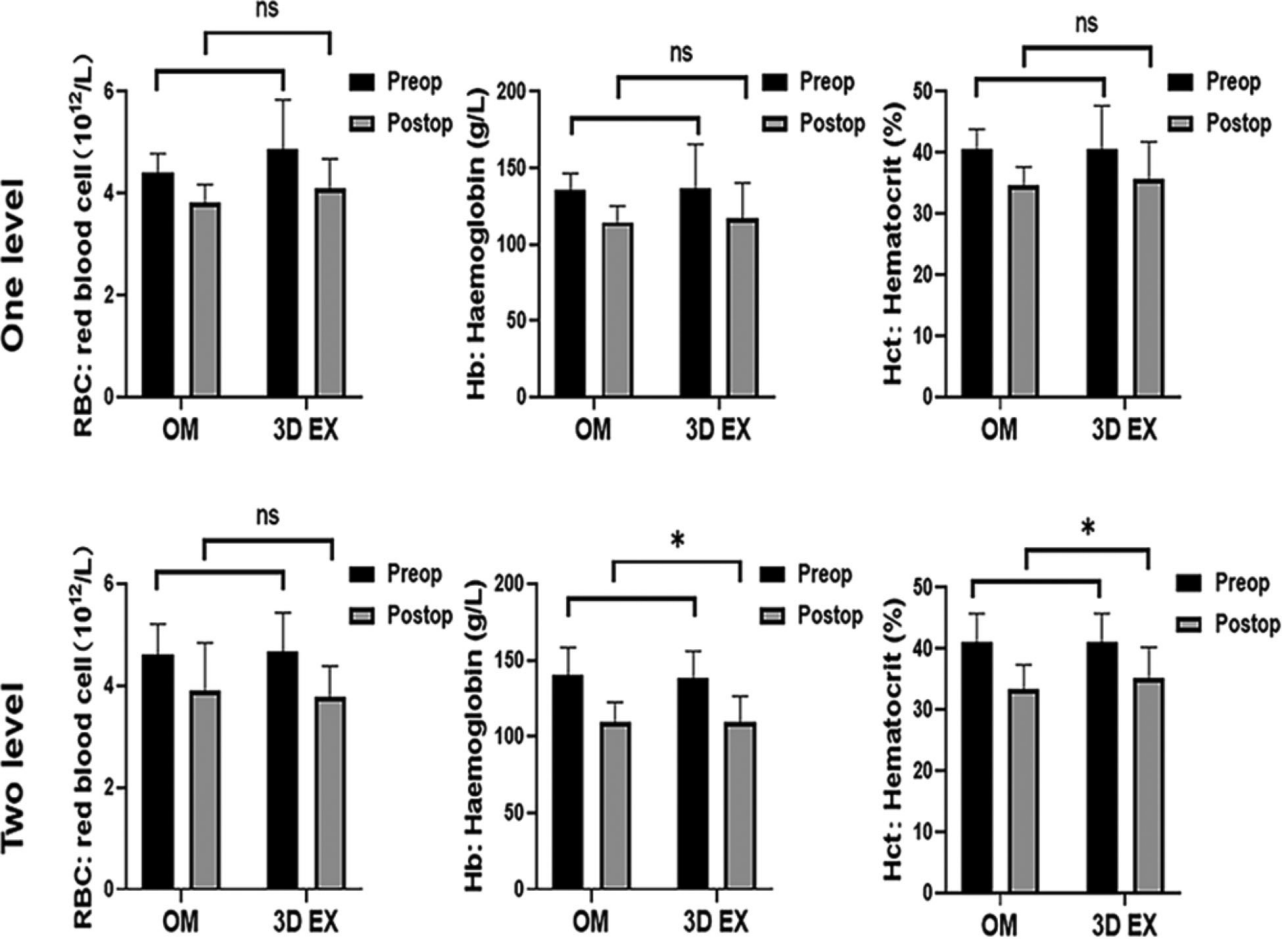
Hematological results. 3D EX, the three-dimensional exoscope; OM, the operating microscope; *p < 0.05; **p < 0.01; ns not statistic difference.

To control baseline imbalances, RBC, Hb, and Hct was adjusted using analysis of covariance tests. RBC levels did not differ significantly between the two groups. There were significant differences in the levels of Hb and Hct values upon using 3D EX, relative to OM for two-level TLIF (*p *= 0.004, *p *= 0.018), but not for one-level TLIF (*p *= 0.533, *p *= 0.140). The two groups did not differ significantly with regards to PBV and HBL. VBL values differed significantly between the two groups. TBL values differed significantly when 3D EX was used for two-level TLIF relative to OM (*p *= 0.046), but not for one-level TLIF (*p *= 0.305). Perioperative blood loss results were shown in Figure 4 and hematological results were shown in Figure 5.

### Clinical outcomes

**Figure 6:**
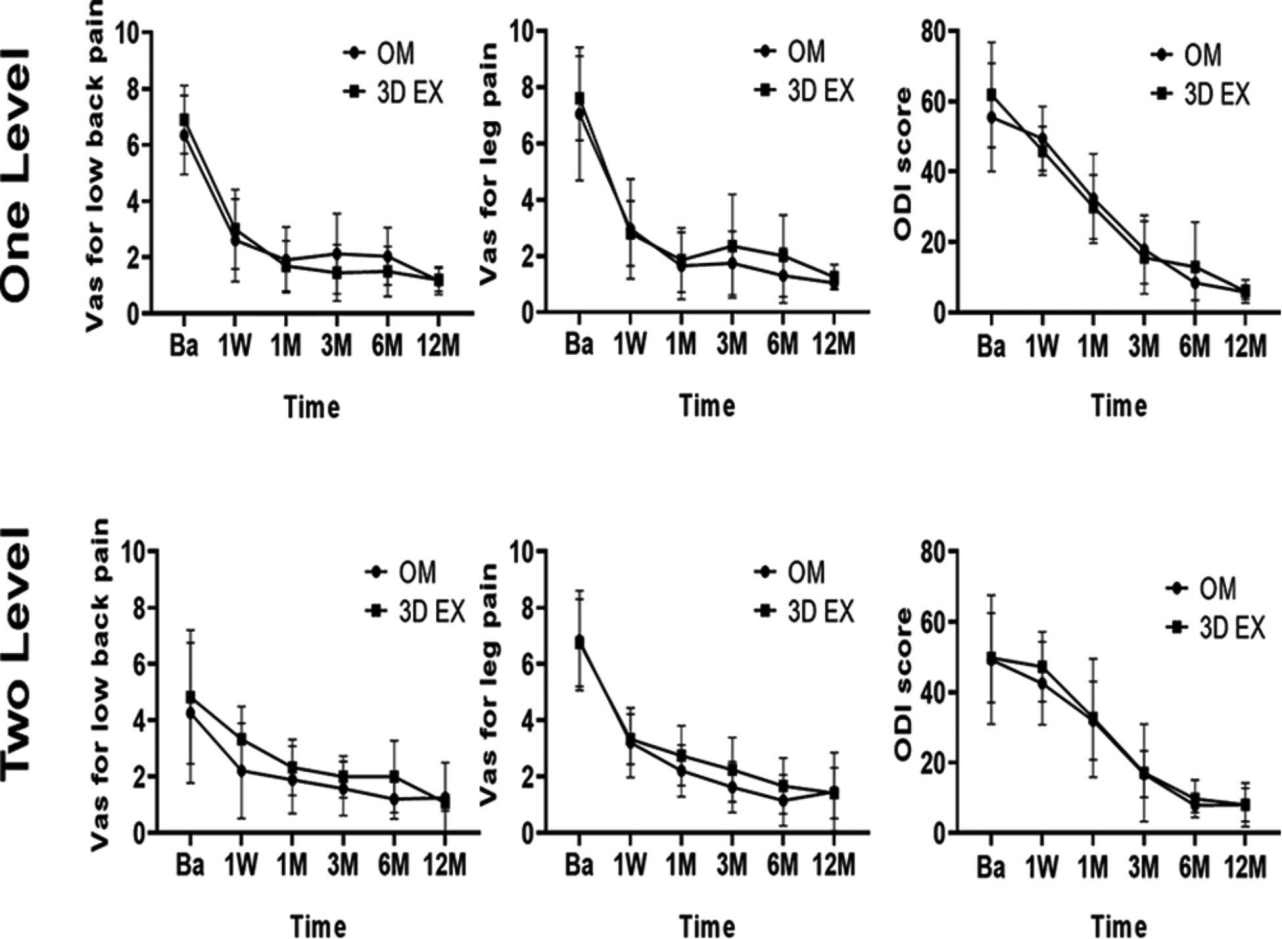
Clinical outcomes. 3D EX, the three-dimensional exoscope; OM, the operating microscope; Ba, baseline; 1W, 1week; 1M, 1month; 3M, 3 months; 6M, 6months; 12M, 12months.

**Figure 7:**
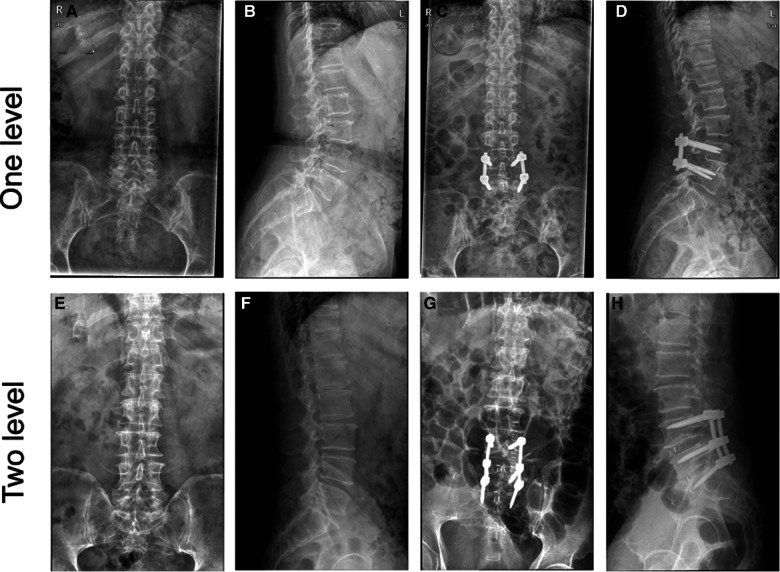
Pre and post-operation 3D TLIF x-rays. (**A**) Preoperative anteroposterior x-ray of one level TLIF. (**B**) Preoperative lateral x-ray of one level TLIF. (**C**) Postperative anteroposterior x-ray of one level TLIF. (**D**) Postperative lateral x-ray of one level TLIF. (**E**) Preoperative anteroposterior x-ray of two level TLIF. (**F**) Preoperative lateral x-ray of two level TLIF. (**G**) Postperative anteroposterior x-ray of two level TLIF. (**H**) Postperative lateral x-ray of two level TLIF.

Postoperative ODI and VAS scores for low back pain, and leg pain were significantly lower than before surgery (all *p *< 0.05). The two groups did not differ significantly with regards to the levels of VAS and ODI at all timepoints (Figure 6). Pre and post-operation 3D TLIF x-rays were shown in Figure 7

## Discussion

The replacement of OM with a high-definition exoscope system in neurosurgery was demonstrated in 2008 (16). Moreover, Ali Shirzadi et al. showed that the video telescope operating monitor (VITOM) system of spinal surgery offers outstanding image quality and an ease of manipulation rivaling OM (17). However, these studies focused on 2D EX only. A major drawback of 2D EX is the lack of stereopsis. In recent years, the development of 3D visualization technology has led to the development of 3D exoscopic visualization which has been used in spinal microsurgery (10, 18–21). However, these reports are mainly based on uncontrolled case reports containing small numbers of subjects, or initial technical reports. There are currently no controlled trials in this area. In this retrospective case-control study, we examined the effect of 3D EX on patients undergoing TLIF.

A shortcoming of past studies was that they only examined intraoperative blood loss during spinal surgery. Zhang et al. found that intraoperative blood loss only explained 36.52% of TBL in TLIF (22). It needed to be emphasized that HBL and postoperative drainage during the perioperative period in TLIF. The main novelties in this paper are the use of drainage data, TBV, TBL, VBL, and HBL. We demonstrate the potential advantages of 3D EX in reducing bleeding by component analysis of perioperative blood loss. What's more, we illuminate directions for perioperative blood management strategies for TLIF.

The most important finding in this article is that 3D EX significantly reduced postoperative drainage volume and perioperative bleeding volume in two-level TLIF relative to OM. Our results show that in two-level TLIF, 3D EX was associated with shorter drainage tube removal time, less total volume of drainage fluid, less use of autologous blood reinfusion, higher postoperative Hb and Hct, and less TBL and VBL relative to the OM group. However, the one level TLIF did not vary as much as in the two levels TLIF. Significant difference could only be found in VBL. Relative to two-level TLIF, one-level TLIF has the advantage of a shorter operation time, less blood loss, and less trauma to patients. Thus, differences were not marked in one-level TLIF.

The following reasons may account for less perioperative bleeding in 3D EX. First, relative to OM, 3D EX is ergonomically improved. Consistent with past findings (19), 3D EX does not confine the surgeon to the eyepieces, reducing fatigue. The wide viewing field and long focal distance of 3D EX offers the surgeon ample space, which may reduce surgical stress, and lower the risk of complications. In operation procedures, wound closure is done only after careful control of the hemostasis at the end of operation. Especially in complex surgeries with long operation time, the surgeons just do not have enough patience and energy for meticulous hemostasis. 3D EX decreases fatigue during long or complicated procedures, enabling surgeons to meticulous hemostasis in the last surgical phase. What's more, the surgeons do not need to switch between microscopic perspective to normal perspective. This make management of surgical instruments for hemostasis more convenient and shallow and deep tissue hemostasis are able to complete at the same time, which promotes intraprocedural hemostasis efficiency. Secondly, the whole surgical team can view the high quality 3D images, which improves the team’s cohesion and training effectiveness (11, 23), as well as improve active participation by the entire team, including nurses and anesthesiologists. For example, once epidural venous plexus bleeding occurs, nurses may also be able to respond more quickly to prepared hemostatic materials and anesthesiologists can provide hypotensive anesthesia to reduce the extent of intraoperative bleeding. Importantly, the wide and clear visual field reduce the need for further microscope adjustments. Especially for intraoperative cage implantation and cage position adjustment, 3D EX eliminates the need for microscope adjustments. Upon laser-guided focusing, clear image of the shallow and deep tissues are simultaneously visible. Thus, this technology can reduce operative time by minimizing the time spent adjusting the microscope position, angle of view, and focus. The superficial and deep hemostasis could conduct at the same time without adjustment.

Various studies have shown that anemia after spinal surgery is associated with increased prolonged hospital stay, postoperative complications, blood transfusion, and mortality (24–26). Our findings show that 3D EX reduces TBL, VBL, and total drainage volume in two-level TLIF, suggesting that patients who undergo 3D EX may have lower incidence of postoperative anemia, which may improve their outcomes. However, we found no intergroup differences with regards to operative time, postoperative hospital stay, and complication rates, which is consistent with past findings (9, 17). This may be due to the small sample size, Another possible explanation is that the TBL of TLIF was too low to reflect advantages of 3D EX. Further studies are needed to determine if 3D EX decreases postoperative hospital stay and complications.

In this retrospective study, both VAS and ODI scores of the two groups improved relative to pre-operative scores and did not differ significantly between the two groups at all timepoints. Because postoperative drainage volume, TBL and VBL did not have a noticeable impact on neurological recovery.

This study has some limitations. First, due to its retrospective design, this study carries inherent deficiencies that may cause confusion or bias. Secondly, all the cases were from a single center and the sample size was small, especially in two-levels TLIF. Thirdly, only cases of lumbar degenerative disc disease were included in the study, with patients with intraspinal tumors excluded. Finally, because our results are based on a single surgical team, they may be challenging to reproduce.

## Conclusion

Here, we show that 3D EX can be used as an alternative to OM in TLIF. Our data show that relative to OM, 3D EX is superior in reducing perioperative bleeding in two-level TLIF. Although the findings should be interpreted with caution, a major strength of this study is that it included perioperative bleeding instead of intraoperative bleeding. Further studies are needed to validate these findings. Our findings highlight 3D EX as an emerging field that will markedly improve microsurgery.

## Data Availability

The original contributions presented in the study are included in the article/Supplementary Material, further inquiries can be directed to the corresponding author/s.
